# Supplementary material for: A binding protein regulates myosin-7a dimerization and actin bundle assembly
Source: Nat Commun. 2021 Jan 25;12:563. doi: 10.1038/s41467-020-20864-z (PMC7835385; doi:10.1038/s41467-020-20864-z)
Supplement: Supplementary file 1 — Supplementary Information [file 41467_2020_20864_MOESM1_ESM.pdf]

Supplementary Figure 1. M7BP binds to myosin-7a with high affinity and activates its ATPase activity.

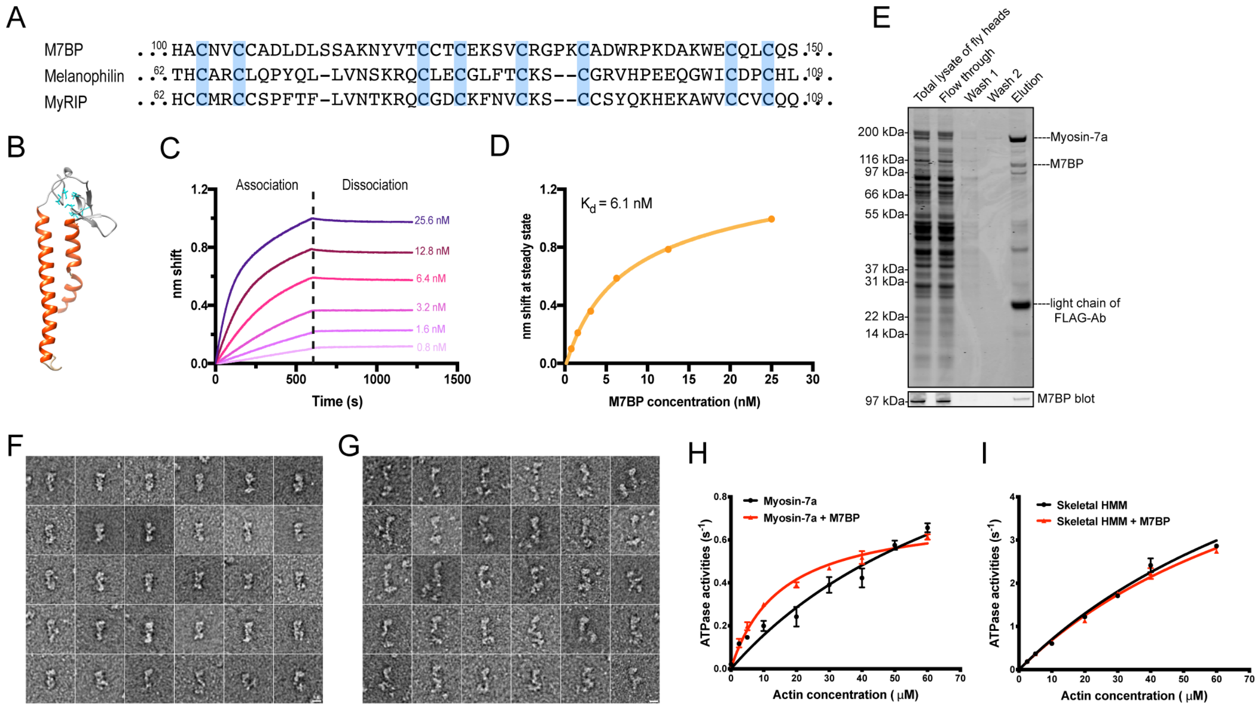

A: Clustal W sequence alignment of the Zn^{2+} binding motifs within Rab-binding domains of M7BP and other Rab-myosin adaptor proteins. The eight conserved cysteine residues responsible for Zn^{2+} binding are highlighted. Numbers indicate the positions of amino acid residues. Sequence used: M7BP (NP_788281.1); Melanophilin (NP_077006.1); MyRIP (NP_056275.2). **B:** Predicted structure of an AA segment (residues 50-170) from the N-terminus of M7BP. Performed with I-TASSER (Iterative Threading ASSEmbly Refinement). Ribbon diagram shows that the predicted structure is consistent with a typical Rab-binding motif which is formed by two α helical regions (colored red) flanking a zinc-binding subdomain (eight conserved cysteine residues for Zn^{2+} binding are shown in blue, as sticks). **C-D:** Bio-layer interferometry (BLI) binding studies with myosin-7a and M7BP. **C**: Time courses of immobilized myosin-7a interacting with increasing concentrations of M7BP. **D:** Steady-state analysis of the

myosin-7a and M7BP binding for equilibrium K_d determination. Data were fitted using nonlinear regression single-site binding. **E:** Affinity pull down assay by using purified myosin-7a protein that was immobilized on FLAG resins to pull out M7BP from the tissue extracts of adult fly heads. Results are representative of 3 independent pull-down assays. **F-G:** Example raw EM images of myosin-7a alone (F) and myosin-7a in complex with M7BP (G). Note that myosin-7a alone (F) typically shows the autoinhibited structure in which the tail fold backs itself and contacts the motor domain. Rarer examples of molecules in which the tail region is extended and not contacting the motor domain are shown to the bottom right of the montage. In the presence of M7BP molecules are typically extended and lack the contact between tail and motor domain. Scale bars = 10 nm. **H-I:** Actin-activated steady-state ATPase assays for myosin-7a (H) and skeletal muscle heavy meromyosin (HMM) (I), in the absence or presence of M7BP. 100 nM myosin-7a, 100 nM M7BP and 20 nM skeletal myosin HMM were used for the experiments. ATPase rates were presented as mean ± SD. N=3 independent experiments. Lines were fit into the Michaelis-Menten equation.

Supplementary Figure 2. Proximity-induced short movement of myosin-7a.

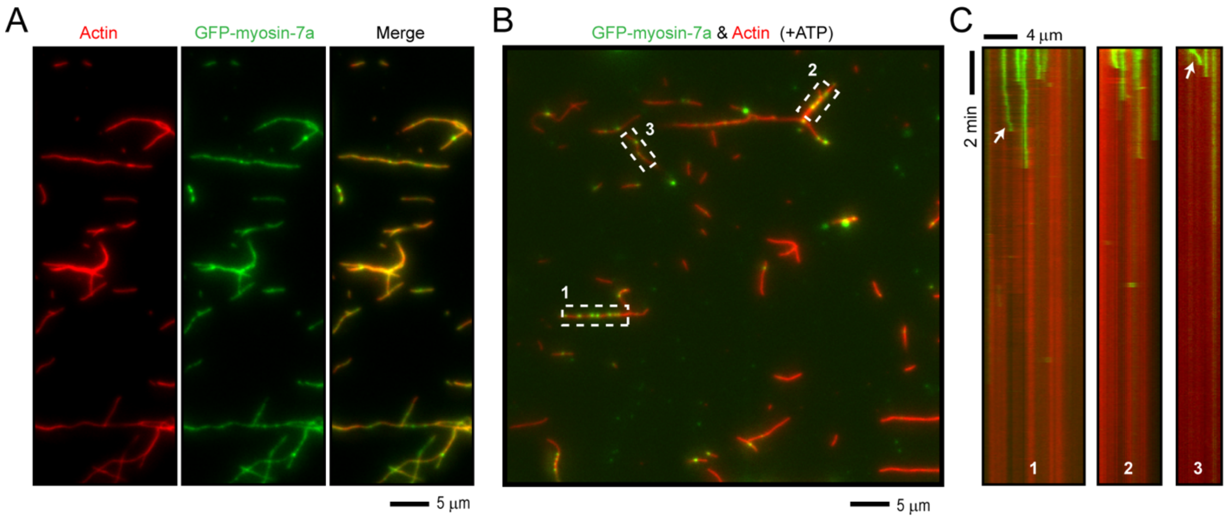

A: GFP-myosin-7a molecules (green) decorate actin filaments (Rhodamine phalloidin labeled, red) in nucleotide-free conditions. **B**: Most myosin-7a were dissociated from actin filaments once ATP was added. **C:** Kymograph analysis of box regions 1, 2, and 3 in B to show examples of short movements of myosin-7a induced by proximity (white arrow). Results are repeated and representative of 3 independent experiments.

Supplementary Figure 3. M7BP-mCherry alone does not interact with actin filaments in single-molecule TIRF assays.

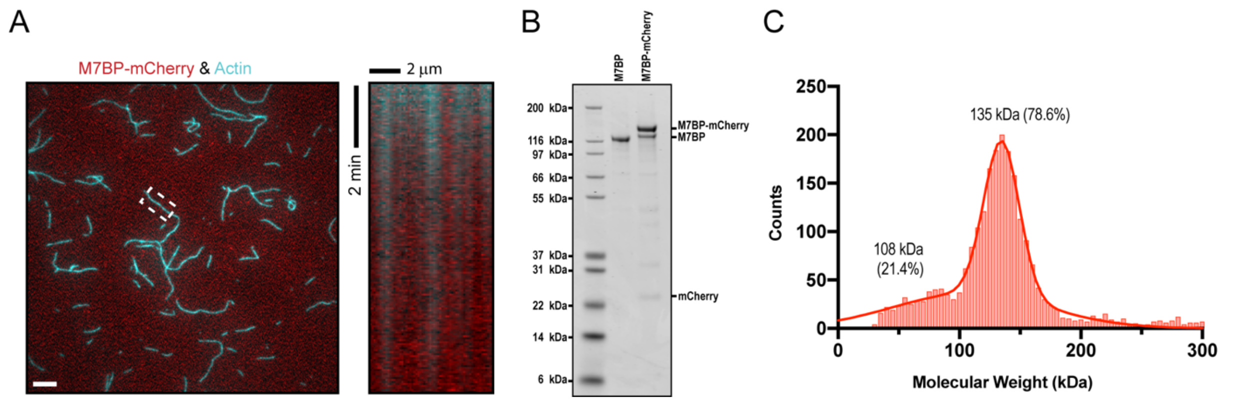

A: Left panel: TIRF field view of 200 nM M7BP (red) with immobilized actin filaments (labeled with Alexa-Fluor 647 phalloidin, cyan) under the buffer same conditions with that in myosin-7a motility assays. **B:** SDS-gels of M7BP and M7BP-mCherry showing partially proteolytic loss of the mCherry tag in M7BP-mCherry fusion protein. **C:** Single-molecule mass photometry analysis of M7BP-mCherry showing that untagged M7BP accounts for 21.4% of total M7BP proteins. N= 2559 protein molecules.

Supplementary Figure 4. Motility characterization of artificially dimerized myosin-7a motors.

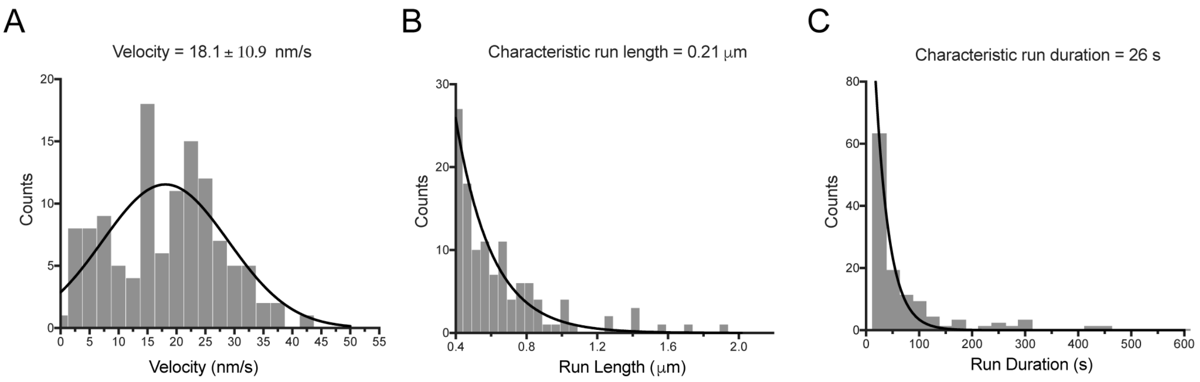

Frequency distribution histograms of velocity (mean ± SD) (A), characteristic run length (B) and characteristic run duration (C) for artificially dimerized myosin-7a motors. N= 119 processive tracks.

Supplementary Figure 5. Myosin-7a and M7BP are both required for inducing filamentous networks in cells.

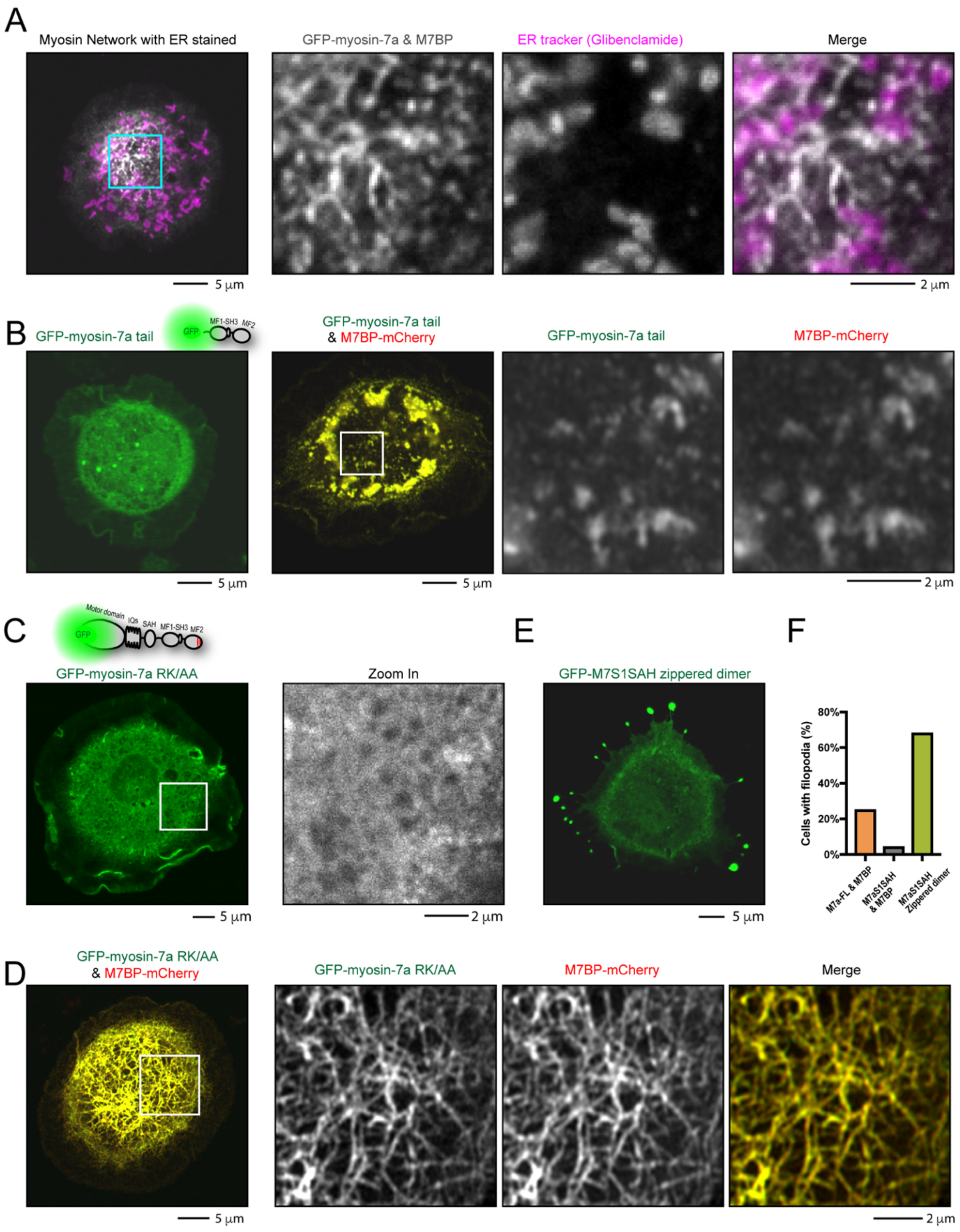

A: Confocal Images of a live *S2* cell expressing myosin-7a-M7BP complexes (visualized by a GFP tag to myosin-7a, grey) with ER membranes labeled with glibenclamide-BODIPY TR (magenta). **B:** Live cell images showing that co-expression of GFP-myosin-7a tail (green) with M7BP-mCherry (red) results in strong colocalization but not filamentous network production. Note that aggregates were formed when the tail-fragment expressed alone and enhanced when it was co-expressed with M7BP. **C:** live cells expressing a myosin-7a mutant RK/AA, which possesses constitutively active ATPase activity, did not develop filopodia or filamentous structures (Airyscan images). **D:** Co-expression of RK/AA mutant (green) with M7BP-mCherry (red) restores the filamentous morphology (Airyscan images). **E:** Cells expressing artificially dimerized myosin-7a motors (green) developed numerous filopodia but not the interior filamentous structures (Confocal images). **F:** Fractions of transfected cells that developed filopodia (in comparison between co-transfection of FL myosin-7a and M7BP (n=40), co-transfection of tailless myosin-7a and M7BP (n=47) and transfection of myosin-7a zippered dimer (n=46)).

Supplementary Table

Primers used in this study

GFP-myosin-7a Forward	5’ GAGCTCGTCGACATGGTGAGCAAGG 3’
GFP-myosin-7a Reverse	5’ CACCATACTAGTCTTGTACAGCTCGTCCAT 3’
Halo-myosin-7a Forward	5’ CGACGAGCTCACTAAATCGGTACTGG 3’
Halo-myosin-7a Reverse	5’ CGATCACCATACTAAATCTCGAGCGT 3’
