# Supplementary material for: A binding protein regulates myosin-7a dimerization and actin bundle assembly
Source: Nat Commun. 2021 Jan 25;12:563. doi: 10.1038/s41467-020-20864-z (PMC7835385; doi:10.1038/s41467-020-20864-z)
Supplement: Supplementary file 3 — Description of Additional Supplementary Files [file 41467_2020_20864_MOESM3_ESM.pdf]

Description of Additional Supplementary Files

File Name: Supplementary Movie 1
Description: Processive Movement of myosin-7a along actin filaments in the presence of M7BP. The movie shows GFP-tagged myosin-7a (green) moving along surface bound actin filaments (Rhodamine phalloidin labeled, red) when M7BP (unlabeled) is present. The movie was collected every 10 seconds for 17 minutes. Scale bar = 5 μm.

File Name: Supplementary Movie 2
Description: Concurrent movement of GFP-myosin-7a and M7BP-mCherry on immobilized actin filaments. Top panel: GFP-myosin-7a alone (green) only transiently associated with actin filaments (Alexa-Fluor 647 phalloidin labeled, blue) without any net motion. Bottom panel: M7BP-mCherry (red) strongly colocalized with GFP-myosin-7a (green) and enabled processive movement of GFP-myosin-7a on actin filaments. Both movies were collected under identical experimental conditions every 3.6 seconds for 10 minutes. Scale bar = 5 μm.

File Name: Supplementary Movie 3
Description: Myosin-7a-M7BP complexes actively drive actin filaments into alignment. Myosin-7a-M7BP complexes (green) move along surface-bound actin filaments (red) and tether free actin filaments (cyan) to align with the immobilized filaments that they were walking on. Scale bar = 5 μm. The movie was collected every 4 seconds for 15 minutes.

File Name: Supplementary Movie 4
Description: Cells expressing myosin-7a-M7BP complexes produce filamentous structures and filopodial protrusions. Myosin-7a and M7BP were labeled with GFP (green) and mCherry (red) tags respectively. Cells were imaged every 8 seconds for 13 minutes. Scale bar = 5 μm.

File Name: Supplementary Movie 5
Description: Myosin-7a-M7BP complexes drive actin filament bundling and filopodia extension. Myosin-7a-M7BP complexes were visualized by a GFP-tag on myosin-7a (green), and the filamentous actin in cells were reported by F-tractin-mCherry (red). Cells were imaged every 10 seconds for 30 minutes. Scale bar = 5 μm.
