# Supplementary material for: A binding protein regulates myosin-7a dimerization and actin bundle assembly
Source: Nat Commun. 2021 Jan 25;12:563. doi: 10.1038/s41467-020-20864-z (PMC7835385; doi:10.1038/s41467-020-20864-z)
Supplement: Supplementary file 9 — Reporting Summary [file 41467_2020_20864_MOESM9_ESM.pdf]

nature research

Corresponding author(s): James R. Sellers

Last updated by author(s): 9/17/2020

Reporting Summary

Nature Research wishes to improve the reproducibility of the work that we publish. This form provides structure for consistency and transparency in reporting. For further information on Nature Research policies, see Authors & Referees and the Editorial Policy Checklist.

Please do not complete any field with "not applicable" or n/a. Refer to the help text for what text to use if an item is not relevant to your study.
For final submission: please carefully check your responses for accuracy; you will not be able to make changes later.

Statistics

For all statistical analyses, confirm that the following items are present in the figure legend, table legend, main text, or Methods section.

n/a	Confirmed	
☐	☒	The exact sample size (*n*) for each experimental group/condition, given as a discrete number and unit of measurement
☐	☒	A statement on whether measurements were taken from distinct samples or whether the same sample was measured repeatedly
☒	☐	The statistical test(s) used AND whether they are one- or two-sided<br>*Only common tests should be described solely by name; describe more complex techniques in the Methods section.*
☒	☐	A description of all covariates tested
☒	☐	A description of any assumptions or corrections, such as tests of normality and adjustment for multiple comparisons
☐	☒	A full description of the statistical parameters including central tendency (e.g. means) or other basic estimates (e.g. regression coefficient) AND variation (e.g. standard deviation) or associated estimates of uncertainty (e.g. confidence intervals)
☒	☐	For null hypothesis testing, the test statistic (e.g. *F*, *t*, *r*) with confidence intervals, effect sizes, degrees of freedom and *P* value noted<br>*Give P values as exact values whenever suitable.*
☒	☐	For Bayesian analysis, information on the choice of priors and Markov chain Monte Carlo settings
☒	☐	For hierarchical and complex designs, identification of the appropriate level for tests and full reporting of outcomes
☒	☐	Estimates of effect sizes (e.g. Cohen's *d*, Pearson's *r*), indicating how they were calculated

Our web collection on statistics for biologists contains articles on many of the points above.

Software and code

Policy information about availability of computer code

Data collection	Nikon TIRF microscopy data were collected using Nikon NIS-Elements Advanced Research (AR) software, version 4.4. Zeiss Confocal and Airyscan data were collected using Zen Black software version 2.3. Electron Microscopy data were collected using AMT Image Capture Engine. Deltavision TIRF-SIM data were collected using SoftWoRx software (Applied Precision). Bio layer interferometry data were collected using Octet software. Cary spectrophotometer data were collected using Cary WinUV software.
Data analysis	Single molecule motility data were analyzed using FIJI (imageJ) with the built in Trackmate plugin. The Trackmate Extras (https://github.com/tinevez/TrackMate-extras) and TrackAnalysis (https://github.com/tinevez/TrackMate-TrackAnalysis) were used to enable output of multi-channel fluorescence intensity and additional run characteristics respectively. Drift correction was applied where necessary using the Image Stabilizer plugin (https://imagej.net/Image_Stabilizer). Electron microscopy data were analysed using SPIDER software (https://spider.wadsworth.org/spider_doc/spider/docs/spider.html). Actin gliding data were analysed using the FAST program (https://github.com/turalaksel/FASTrack). Data throughout the study were analyzed using Graphpad Prism 7.0.

For manuscripts utilizing custom algorithms or software that are central to the research but not yet described in published literature, software must be made available to editors/reviewers. We strongly encourage code deposition in a community repository (e.g. GitHub). See the Nature Research guidelines for submitting code & software for further information.

Data

Policy information about availability of data

All manuscripts must include a data availability statement. This statement should provide the following information, where applicable:
- Accession codes, unique identifiers, or web links for publicly available datasets
- A list of figures that have associated raw data
- A description of any restrictions on data availability

All primary data ~~and customized code~~ used in this manuscript are available from the authors upon request.

Field-specific reporting

Please select the one below that is the best fit for your research. If you are not sure, read the appropriate sections before making your selection.

☒ Life sciences ☐ Behavioural & social sciences ☐ Ecological, evolutionary & environmental sciences

For a reference copy of the document with all sections, see nature.com/documents/nr-reporting-summary-flat.pdf

Life sciences study design

All studies must disclose on these points even when the disclosure is negative.

Sample size	Sample sizes were not calculated prior to experiments as statistical comparison between experimental groups was not part of the experimental design. Sample sizes for experiments were chosen to be as large as possible whilst producing datasets of a practical size for use in subsequent analysis.
Data exclusions	As described in the experimental procedures, electron microscopy data were subjected to two rounds of alignment and classification with poorly aligned particles removed after the first round. The criteria for removal were a lack of distinct features in the class averages due to the grouping of dissimilar images (typically a circular appearance with poorly defined and blurred edges). In actin gliding analyses, a 33% tolerance filter was used to remove data from filaments in which the standard deviation of filament velocity was greater than 33% of the mean filament velocity and filaments with velocity <0.1 nm/s were also excluded. These filters were decided upon prior to analysis and are commonly used in the field for this type of analysis. They exclude the small number of filaments which are either moving intermittently or stuck to inactive myosin heads on the surface, which would otherwise contribute to the calculated mean velocity.
Replication	Experimental replicates were performed where possible and practical as indicated in the text. The results from single molecule experiments were reproduced across multiple protein preparations including comparisons with and without additional fluorophores tags etc and are expected to be highly reproducble by other researchers. The phenotypes seen in expression studies in cells were reproducible and the same effects were observed in different experiments using different imaging modalities.
Randomization	Data were not randomized as statistical comparison between experimental groups was not part of the experimental design.
Blinding	Investigators were not blinded to group allocation as statistical comparison between experimental groups was not part of the experimental design.

Reporting for specific materials, systems and methods

We require information from authors about some types of materials, experimental systems and methods used in many studies. Here, indicate whether each material, system or method listed is relevant to your study. If you are not sure if a list item applies to your research, read the appropriate section before selecting a response.

Materials & experimental systems

n/a	Involved in the study	
☐	☒	Antibodies
☐	☒	Eukaryotic cell lines
☒	☐	Palaeontology
☒	☐	Animals and other organisms
☒	☐	Human research participants
☒	☐	Clinical data

Methods

n/a	Involved in the study	
☒	☐	ChIP-seq
☒	☐	Flow cytometry
☒	☐	MRI-based neuroimaging

Antibodies

Antibodies used	Biotinylated GFP antibody (Rockland, 600-406-215); Myosin-7a antibody (produced with Invitrogen); M7BP antibody (produced with SDIX)
Validation	Biotinylated GFP antibody (ELISA; IF; Western Blot; IHC). Myosin-7a antibody (Drosophila, IF). M7BP antibody (Drosophila, IF)

Eukaryotic cell lines

Policy information about cell lines

Cell line source(s)	Drosophila S2 cells (Gibco)
Authentication	Cell lines were tested for contamination of bacteria, yeast, mycoplasma and virus and was characterized by isozyme and karyotype analysis.

Mycoplasma contamination	All cell lines were tested negative for mycoplasma contamination.
Commonly misidentified lines (See ICLAC register)	None.

This checklist template is licensed under a Creative Commons Attribution 4.0 International License, which permits use, sharing, adaptation, distribution and reproduction in any medium or format, as long as you give appropriate credit to the original author(s) and the source, provide a link to the Creative Commons license, and indicate if changes were made. The images or other third party material in this article are included in the article's Creative Commons license, unless indicated otherwise in a credit line to the material. If material is not included in the article's Creative Commons license and your intended use is not permitted by statutory regulation or exceeds the permitted use, you will need to obtain permission directly from the copyright holder. To view a copy of this license, visit http://creativecommons.org/licenses/by/4.0/
